# Safety and efficacy of ultrathin strut biodegradable polymer sirolimus-eluting stent versus durable polymer drug-eluting stents: a meta-analysis of randomized trials

**DOI:** 10.1186/s12872-018-0902-5

**Published:** 2018-08-15

**Authors:** Ping Zhu, Xin Zhou, Chenliang Zhang, Huakang Li, Zhihui Zhang, Zhiyuan Song

**Affiliations:** Department of Cardiology, Southwest Hospital, Third Military Medical University (Army Medical University), Chongqing, China

**Keywords:** Meta-analysis, Biodegradable polymer, Durable polymer, Percutaneous coronary intervention

## Abstract

**Background:**

The Orsiro biodegradable polymer sirolimus-eluting stent (O-SES) is a new-generation biodegradable polymer drug-eluting stent with the thinnest strut thickness to date developed to improve the percutaneous treatment of patients with coronary artery disease. We perform a meta-analysis of randomized clinical trials (RCTs) comparing the efficacy and safety of an ultra-thin, Orsiro biodegradable polymer sirolimus-eluting stent (O-SES) compared with durable polymer drug-eluting stents (DP-DESs).

**Methods:**

Medline, Embase, and CENTRAL databases were searched for randomized controlled trials comparing the safety and efficacy of O-SES versus DP-DES. Paired reviewers independently screened citations, assessed risk of bias of included studies, and extracted data. We used the Mantel-Haenszel method to calculate risk ratio (RR) by means of a random-effects model.

**Results:**

Six RCTs with a total of 6949 patients were selected. All included trials were rated as low risk of bias. The O-SES significantly reduced the risk of myocardial infarction (RR 0.78, 95% confidence interval [CI] 0.62–0.98; I^2^ = 0%; 10 fewer per 1000 [from 1 fewer to 18 fewer]; high quality) compared with the DP-DES. There was no significant difference between O-SES and DP-DES in the prevention of stent thrombosis (RR: 0.75; 95% CI: 0.52–1.08), cardiac death (RR: 0.93; 95% CI: 0.63–1.36), target lesion revascularization (RR 1.10, 95% CI 0.86–1.42) and target vessel revascularization (RR 0.97, 95% CI 0.78–1.21).

**Conclusion:**

Among patients undergoing percutaneous coronary intervention, O-SES resulted in significantly lower rates of myocardial infarction than DP-DES and had a trend toward reduction in stent thrombosis.

## Background

The implantation of a drug-eluting stent (DES) that prevent restenosis by the release of antiproliferative agents from polymers is considered the standard approach for percutaneous coronary intervention [1]. After DES implantation, however, the lifelong presence of a durable polymer (DP) might induce chronic inflammation, cell proliferation, delay arterial healing, long-term endothelial dysfunction, and occasionally cause cardiovascular events such as myocardial infarction (MI) and stent thrombosis (ST) [2, 3]. Raising awareness of this risk motivated the improvements of stents with biodegradable polymer (BP) allowing elimination of the polymer by degradation. Despite these iterations, the potential benefits for BP-DES remain largely unproven. BP-DES has shown superior profiles over bare-metal stents and first-generation DP-DES [4–6] but shares a similar efficacy and safety profile compared with second-generation DP-DES [7, 8].

The Orsiro biodegradable polymer sirolimus-eluting stent (O-SES; Biotronik, Bülach, Switzerland) is a novel DES consisting of an ultrathin strut cobalt chromium design with a bioresorbable, poly-Llactic acid polymer coating that releases sirolimus [9]. Furthermore, O-SES has the thinnest strut thickness to date (60 μm), and thus provides good flexibility and deliverability. Preclinical study has reported that thin struts reduced both intimal proliferation and thrombus formation [10]. Evidence in the bare-metal stent era suggested reduced arterial injury and angiographic restenosis with low stent strut thickness [11]. The reduced strut thickness of 40% has been reported to improve outcomes compared with early generation drug-eluting stents [12]. Thus, the use of thin struts might reduce the risk of potentially fatal complications, such as ST and MI [10].

Recently, the safety and efficacy of O-SES compared with contemporary DP-DES has been assessed in randomized controlled trials (RCTs) [13–18]. However, the results of these trials were controversial. Early, modest-sized studies in this field failed individually to prove that O-SES was super to DP-DES [13–15, 17, 18]. In late 2017, a new trial has endorsed the safety and effectiveness of O-SES compared with DP-everolimus-eluting stents (EES) [16]. Therefore, we conducted a meta-analysis to compare the efficacy and safety of O-SES to DP-DES.

## Methods

The registered study protocol is available on PROSPERO (CRD42017081107). The findings of the meta-analysis was reported according to the Preferred Reporting Items for Systematic Reviews and Meta-Analyses (PRISMA) [19].

### Eligibility criteria

#### Inclusion criteria

1. Population: adult participants (≥18 years) with percutaneous coronary intervention.
2. Intervention: percutaneous coronary intervention with O-SES.
3. Comparison intervention: percutaneous coronary intervention with DP-SES.
4. Outcome: Primary outcome was MI, as defined by the individual trials. Secondary outcomes were definite or probable ST, cardiac death, target vessel revascularization (TVR), and target lesion revascularization (TLR).
5. Study design: RCT.

#### Exclusion criteria

We excluded duplicate reports and post hoc analyses.

### Search strategy

Medline, EMBASE, and the Cochrane Library at the CENTRAL Register of Controlled Trials were searched with the assistance of a professional librarian. The last electronic search was performed on October 20, 2017. We also reviewed the reference lists of the original trials, prior meta-analysis, and review articles. There were no restrictions on language. For the search strategy, we used, in various relevant combinations, MeSH terms and keywords pertinent to the intervention of interest: “biodegradable polymer”, “Orsiro”, “drug-eluting stent”, “sirolimus”, “durable polymer”, “controlled trials” and “randomized controlled trial.” (Table 3 in Appendix 1).

### Study selection

Two investigators performed the study selection independently. They screened titles and abstracts for initial study inclusion. They screened the full text of potentially relevant trials. Disagreements were resolved by consensus with a senior author. Follow-up of all outcomes was at 12 months.

### Data collection process

Two investigators independently extracted data from the included RCTs using a standardized electronic form. Disagreements between the two investigators were resolved by consensus with a third investigator. Authors of studies were contacted when suitable data were not available.

### Assessment of risk of bias and quality of evidence

Two investigators assessed the risk of bias of the trials by using the risk of bias tool of The Cochrane Collaboration [20]. Disagreements were discussed with a third author. Trials with more than two high-risk components were considered as a moderate risk of bias, and trials with more than four high-risk components as having a high risk of bias.

We used the GRADE approach to rate the quality of evidence and generate absolute estimates of effect for the outcomes [21]. We used detailed GRADE guidance to assess the overall risk of bias, indirectness, inconsistency, imprecision and publication bias and summarized results in an evidence profile.

### Outcomes

The safety outcomes of the analysis included MI, definite or probable ST, and cardiac death, and the efficacy outcomes included TVR and TLR. The primary outcome was MI, which was defined by the individual trials.

### Data synthesis

Computations were performed with RevMan- v 5.3.3 (a freeware available from The Cochrane Collaboration). Analyses for all outcomes were done on an intention-to-treat basis. The meta-analysis was done using random effect models regardless of the level of heterogeneity. The risk ratios (RR) along with 95% confidence intervals (CI) was calculated for dichotomous data. We assessed heterogeneity with the Chi^2^ test (threshold *p* = 0.10) and the I^2^ tests, I^2^ values lower than 25%, 25–50%, and higher than 75% represented low, moderate, and high heterogeneity, respectively [22]. A 2-tailed *P* value of < 0.05 was set for statistical significance.

We conducted trial sequential analysis (TSA) for primary outcome (MI) using TSA software (version 0.9.5.9; Copenhagen Trial Unit, Copenhagen, Denmark) [23]. We used the O’Brien-Fleming approach to compute the trial sequential monitoring boundaries. An optimal information size was set to a two-sided alpha of 0·05, beta 0·80, relative risk reduction of 20%.

If a pooled analysis included 10 or more studies, we planned to use a funnel plot to explore the possibility of published bias.

We performed a subgroup analysis according to the different types of DP-DES (Everolimus versus Zotarolimus).

We planned sensitivity analyses:1. by performing meta-analysis using both fixed-effect models; 2. using alternative imputation methods; 3 using odds ratios instead of risk ratios;

## Results

### Study selection and characteristics

**Fig. 1:**
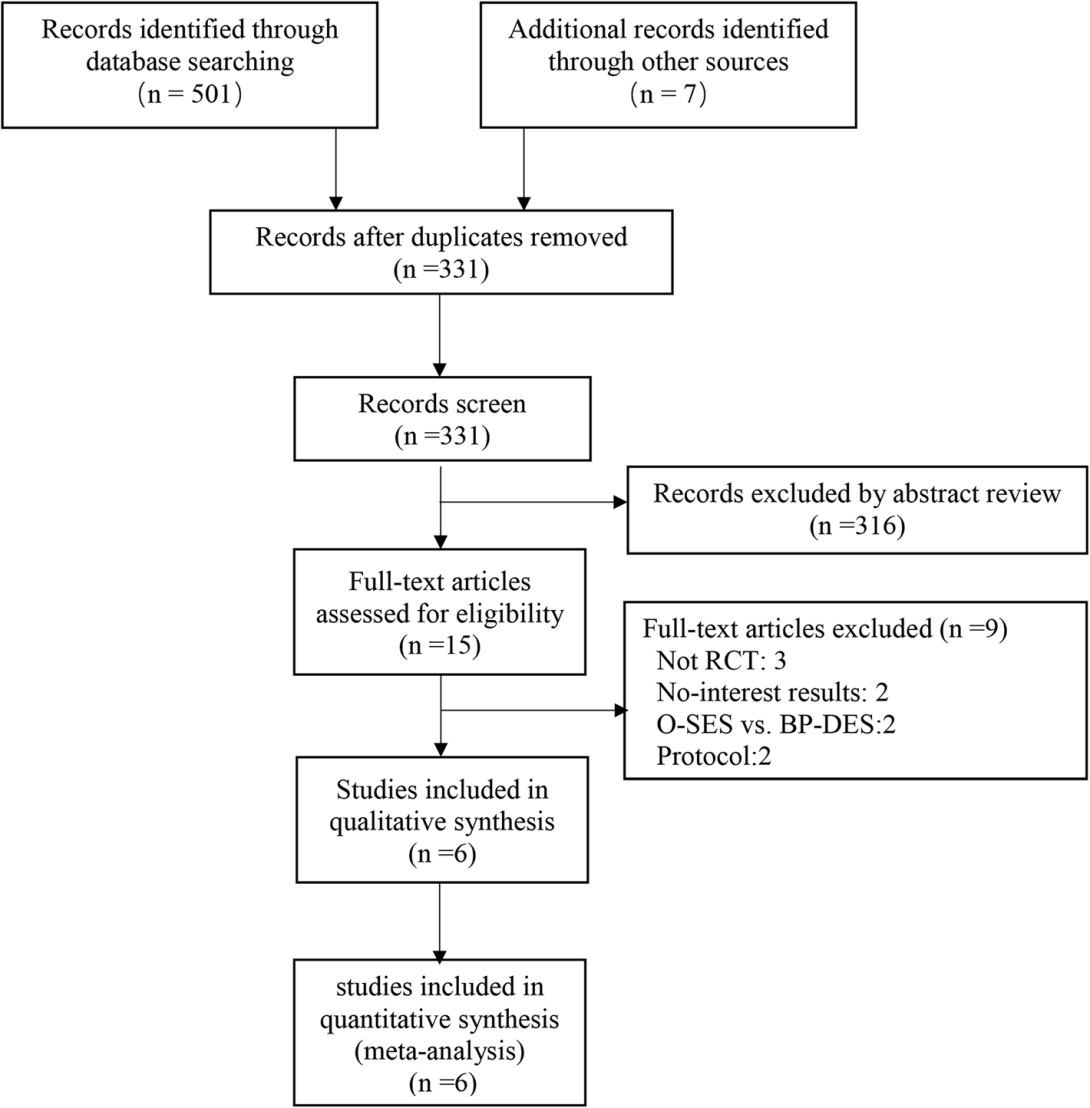
Search strategy and final included and excluded studies

The search strategy yielded 331 manuscript abstracts (Fig. 1). Excluding 316 non-pertinent titles or abstracts, 15 studies were assessed according to the selection criteria. Six trials [13–18] were included in the meta-analysis.

### Study characteristics

The baseline characteristics of included trials have been summarized in Table 1 and Tables 4 and 5 in Appendices 2 and 3. All trials published from 2015 to 2017. A total of 3120 patients receiving DP-SES compared with 3829 patients treated with O-SES. The types of DP-DES included zotarolimus-eluting stents (ZES, 2 trials) and everolimus-eluting stents (EES, 4 trials). All trials reported outcomes at 12-months follow-up, whereas one [15] of them even reported outcomes at 24-months follow-up. To decrease heterogeneity, we included only outcomes at 12-months follow-up in the meta-analysis.

**Table 1:** Characteristics of patients in eligible studies

Trial	Year	No. of Patients		Follow-up (months)	DAPT (Months)	O-DES Characteristics			DP-DES Characteristics		
		O-SES	DP-DES			Stent	Thickness	Drug	Stent	Thickness	Drug
BIO-RESORT	2016	1169	1173	12	6	Orsiro	60	Sirolimus	Resolute Integrity	91	zotarolimus
BIOFLOW II	2015	298	154	12	> 6	Orsiro	60	Sirolimus	Xience Prime	81	Everolimus
BIOFLOW V	2017	884	450	12	> 6	Orsiro	60	Sirolimus	Xience Prime	81	Everolimus
BIOSCIENCE	2016	1063	1056	12	12	Orsiro	60	Sirolimus	Xience Prime	81	Everolimus
ORIENT	2017	250	122	12	> 12	Orsiro	60	Sirolimus	Resolute Integrity	91	zotarolimus
PRISON IV	2017	165	165	12	> 12	Orsiro	60	Sirolimus	Xience Prime	81	Everolimus

### Risk of bias and quality of evidence

**Fig. 2:**
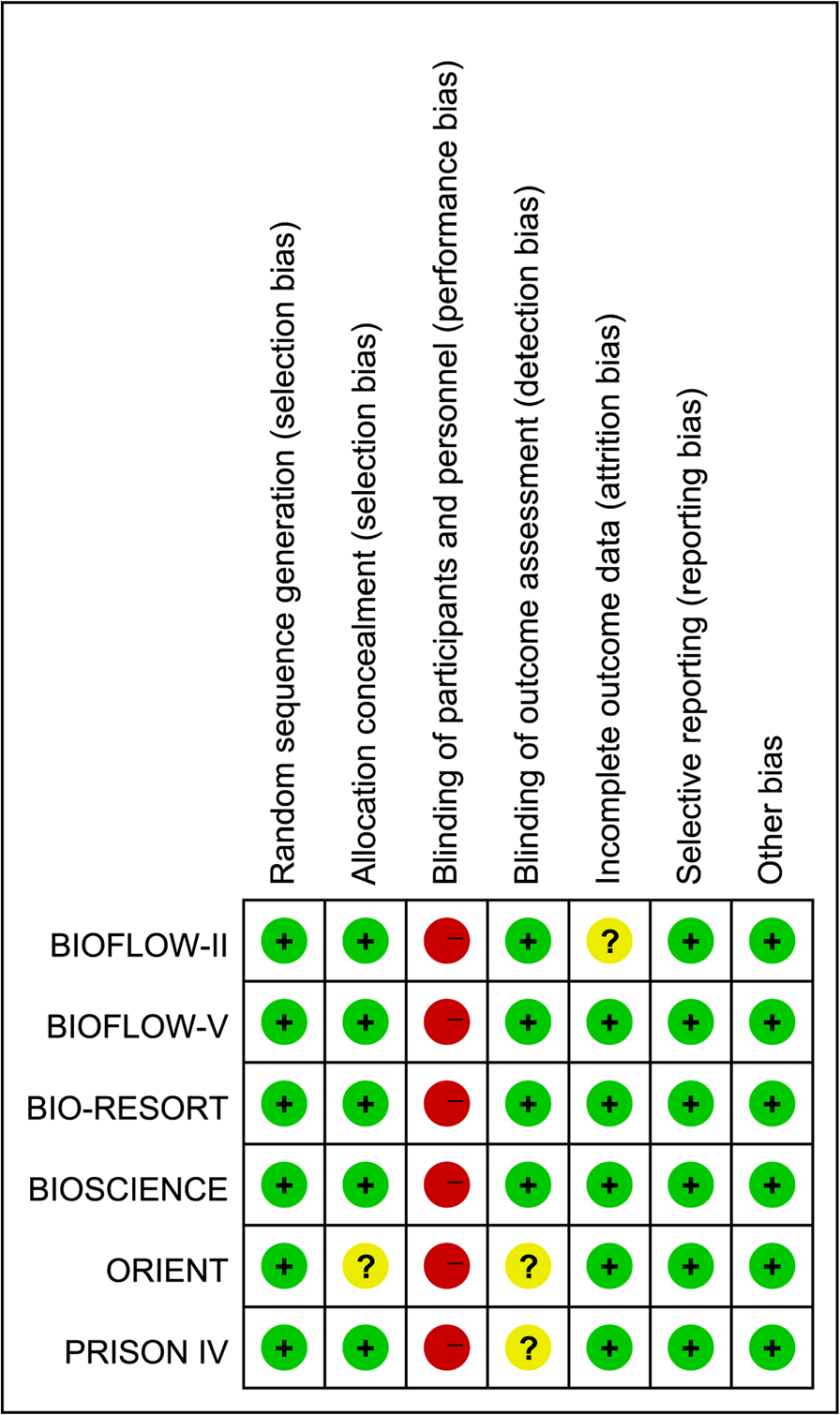
Risk of bias summary

All six trials were at low risk of bias (Fig. 2). The greatest risk of bias came from blinding. The nature of the trial interventions precluded blinding of their physicians; whereas five of trials stated that blinding of outcome assessment was used and the other one was unclear. GRADE summary findings for all outcomes is showed in Table 2. We did not use funnel plots to assess the existence of possible publication bias because there were only six trials included in our meta-analysis.

**Table 2:** GRADE evidence profile of outcomes, O-SES versus DP-DES

Outcome	No. of patients (Studies)		Study results (95% CI) and measurements	Absolute effect estimates (per 1000)			Quality	Importance
	O-SES	DP-DES		O-SES	DP-DES	Absolute Risk (95% CI)		
Myocardial infarction	142/3777 (3.8%)	147/3095 (4.7%)	RR 0.78 (0.62 to 0.98)	37	47	10 fewer (from 1 fewer to 18 fewer)	⊕ ⊕ ⊕ ⊕ High	Critical
Stent thrombosis	50/3767 (1.3%)	63/3095 (2%)	RR 0.75 (0.52 to 1.08)	15	20	5 fewer (from 10 fewer to 2 more)	⊕ ⊕ ⊕⊝ Moderate^a^	Important
Cardiac death	50/3777 (1.3%)	50/3095 (1.6%)	RR 0.93 (0.63 to 1.36)	15	16	1 fewer (from 6 fewer to 6 more)	⊕ ⊕ ⊕⊝ Moderate^a^	Important
Target vessel revascularization	166/3778 (4.4%)	141/3092 (4.6%)	RR 0.97 (0.78 to 1.21)	45	46	1 fewer (from 10 fewer to 10 more)	⊕ ⊕ ⊕ ⊕ High	Important
Target lesion revascularization	129/3777 (3.4%)	101/3094 (3.3%)	RR 1.1 (0.86 to 1.42)	36	33	3 more (from 5 fewer to 14 more)	⊕ ⊕ ⊕ ⊕ High	Important

*CI* Confidence interval, *RR* Risk ratio, *O-SES* Orsiro biodegradable polymer sirolimus-eluting stent, *DP-DES* Durable polymer drug-eluting stents;

High quality: Further research is very unlikely to change our confidence in the estimate of effect. Moderate quality: Further research is likely to have an important impact on our confidence in the estimate of effect and may change the estimate. Low quality: Further research is very likely to have an important impact on our confidence in the estimate of effect and is likely to change the estimate. Very low quality: We are very uncertain about the estimate

^a^Serious imprecision

### Safety endpoints: MI, ST, and cardiac mortality

**Fig. 3:**
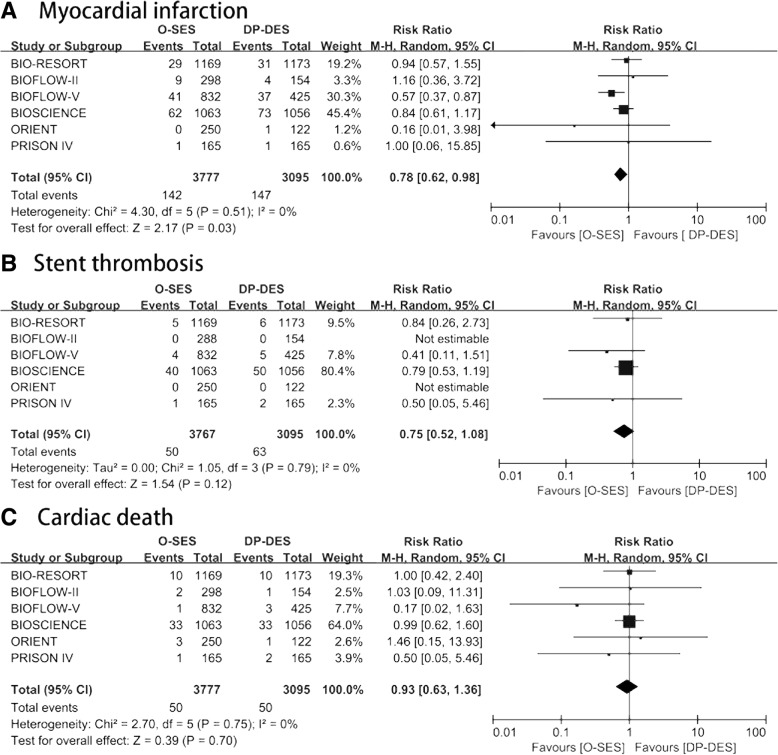
Forest plot assessing safety outcomes. A: myocardial infarction, B: definite or probable stent thrombosis, C: cardiac death. CI = confidence interval; M-H = Mantel-Haenszel; SE = standard error

The associations between O-SES versus DP-DES and safety outcomes are shown in Fig. 3. All six trials reported safety outcomes. MI occurred in 142 of 3777(3.8%) participants randomized to the O-SES group and 147 of 3095(4.8%) participants randomized to the medical therapy group. The risk ratio (RR) for MI also confer an advantage of O-SES over DP-DES (RR 0.78, 95% CI 0.62–0.98; I^2^ = 0%; 10 fewer per 1000 [from 1 fewer to 18 fewer]; high quality). Sensitivity analyses using an alternative statistical method (Inverse Variance; RR 0.78, 95% CI 0.62–0.97; I^2^ = 0%), effect measure (Odds Ratio 0.77, 95% CI 0.60–0.97; I^2^ = 0%), and analysis model showed similar results of MI (Fixed; RR 0.78, 95% CI 0.62–0.98; I^2^ = 0%). TSA confirmed that the required information size was not met (Fig. 5 in Appendix 4).

The meta-analysis showed no significant difference between O-SES and DP-SES on ST (RR: 0.75; 95% CI: 0.52–1.08; I^2^ = 0%) or cardiac mortality (RR: 0.93; 95% CI: 0.63–1.36; I^2^ = 0%).

### Efficacy outcomes: TVR and TLR

**Fig. 4:**
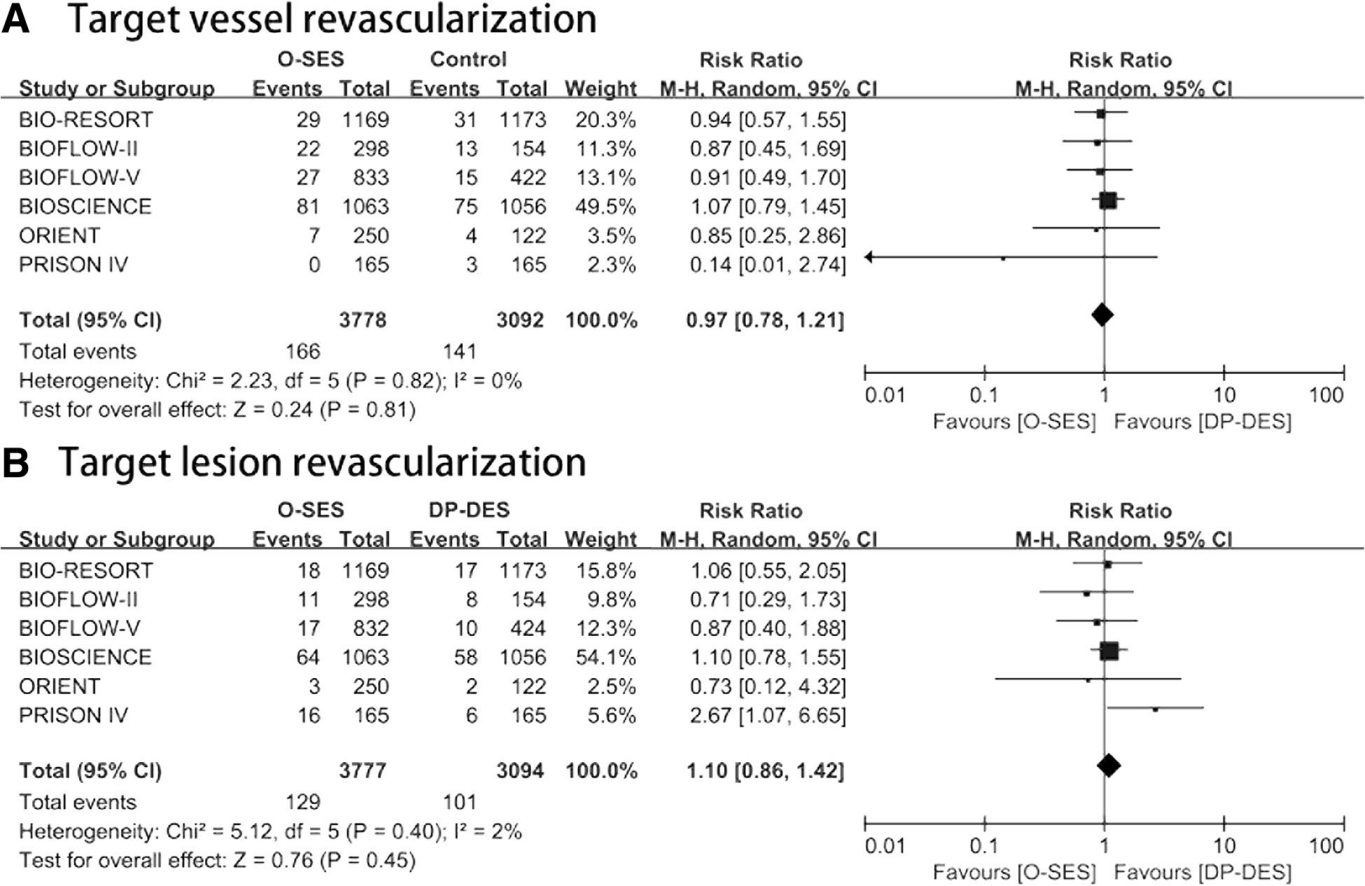
Forest plot assessing efficacy outcomes. A: target vessel revascularization, B: target lesion revascularization (TLR). CI = confidence interval; M-H = Mantel-Haenszel; SE = standard error

**Fig. 5:**
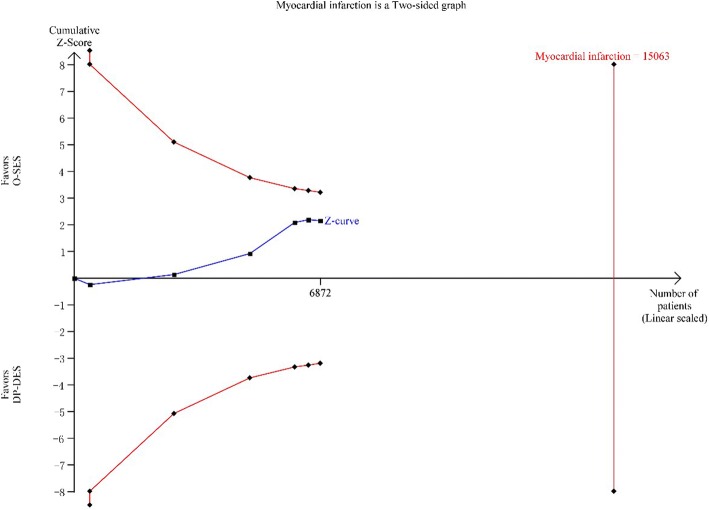
Trial sequential analysis of myocardial infarction

All the included studies presented outcomes of TVR and TLR, showing that there was no statistically significant difference between O-SES and DP-DES regarding TVR (RR 0.97, 95% CI 0.78–1.21) and TLR (RR 1.10, 95% CI 0.86–1.42). (Fig. 4).

### Subgroup analysis

**Fig. 6:**
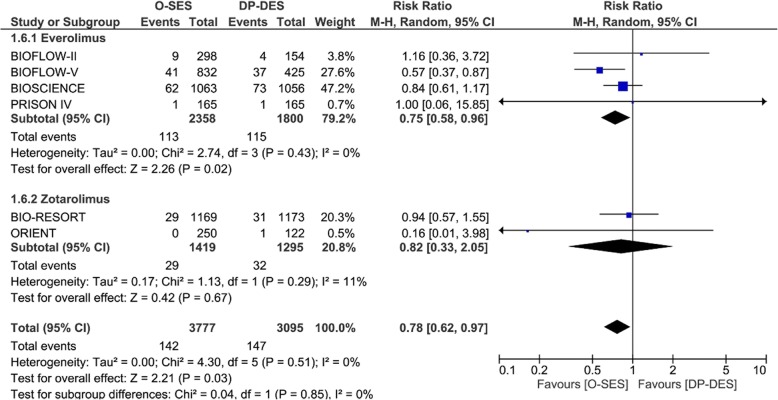
Forest plot assessing myocardial infarction of subgroup analysis based on various DES types (everolimus and zotarolimus)

We performed a subgroup analysis based on various DES types (everolimus and zotarolimus). Like the overall analysis, this subgroup analysis showed that O-SES has certain benefit in reducing risk of MI compared to DP-EES (RR 0.75, 95% CI 0.58–0.96; I^2^ = 0%, Fig. 6 in Appendix 5) but this benefit did not show in cardiac mortality, ST, TVR, or TLR. There is no significant difference between O-SES and DP-ZES in the risks of MI, cardiac mortality, TVR, or TLR.

## Discussion

In this meta-analysis of 6 RCTs, we found that MI was significantly lower in patients with O-SES than in patients with DP-DES. There was no evidence of a difference between groups concerning cardiac mortality, ST, TLR, and TVR.

Possibly our most important finding was the significant risk reduction for MI in patients with O-SES compared with DP-DES. Contrary to our meta-analysis, however, recent meta-analyses showed that BP-DES were similar regarding cardiovascular outcomes including MI compared to second-generation DP-DES [8, 24]. Similarly, a meta-analysis comparing BP-SES with DP-DES found there was no significant difference in the risk of MI [25]. The different results regarding MI between our meta-analysis and previous meta-analyses may be explained by the different eligibility criteria. Our meta-analysis included trials comparing O-SES with DP-DES rather than BP-DES (or BP-SES) with DP-DES. O-SES has the thinnest strut thickness to date. It is probable that the thinner stent struts of the O-SES (60 μm) compared with DP-DES (81–91 μm) lead to the lower risk of MI. The effect of stent strut thickness has been well established. In fact, compared to the thicker struts, thinner struts have been shown to reduce vessel injury, inflammation, neointimal proliferation, and thrombus formation [10, 11, 26, 27]. Reduction in strut thickness from stainless steel (132–140 μm) to chromium alloys (81–91 μm) contributed to a decreased risk of MI by about 40–80% [28–31].

Our meta-analysis has reported results suggestive of a protective effect of O-SES on ST compared with DP-DSE but failed to show the statistical significance of this association. One explanation for this fail was the small number of events during the follow-up. New generation DES have the most favourable safety and efficacy outcomes to date, adverse events have become less frequent in the past decade. Thus, to find a significant difference in management strategy, additional RCTs needs to follow patients for a long duration or enrol substantial numbers.

Our study did not show a significant decrease of TVR or TLR in O-SES compared with DP-DES. Indeed, two prior network meta-analyses have demonstrated a reduced risk of TVR and TLR of BP-DES compared to DP-DES [4, 32]. But, BP-SES were not included in the two network meta-analyses. A meta-analysis comparing BP-SES with DP-DES found similar efficacy profiles between those groups [25].

### Strengths and limitations

Strengths of our meta-analysis included duplicate assessment of risk of bias, eligibility, and data abstraction. The meta-analysis included a rigorous assessment of the quality of evidence. We have evaluated relative and absolute risks, which are crucial for making decisions between O-SES and DP-DES.

First, different DP-DES platforms were used for comparison in the RCTs included in our meta-analysis. However, authors attempted to overcome these differences by performing a subgroup analysis based on DP-DES. We found O-SES significantly decreased MI compared to DP-EES but not to DP-ZES.

Second, a small number (6 RCTs, 6949 patients) and short follow-up duration (12 months) of included trials might afford insufficient ability to detect differences in rare events. For example, our results might suggest a reduced ST in O-SES but failed to show the statistical significance of this association. Thus, longer duration of follow-up and larger populations are required for further research.

Third, the limited number of included RCTs lead to insufficiently detect the presence of publication bias. However, publication bias is unlikely as most included RCTs had negative results.

Fourth, previous meta-analysis suggested a possible increased midterm risk for ST and MI with BP-DES [33]. However, we did not report mid- and long-term outcomes in this topic, because follow-up data of longer than 1 year is limited. Thus, the mid- and long-term safety and efficacy of O-SES vs. DP-DES is not clealy established.

Fifth, although the statistical heterogeneity was very low in most outcomes (I^2^ = 0), there may be substantially clinical heterogeneity, which was driven by differences in methodological and clinical features between trials. For example, the duration and the type of dual antiplatelet therapy may have an influence on outcomes; however, we cannot perform a subgroup analyses on dual antiplatelet therapy because of lack of data from included trials. Sixth, TSA found that the required information size was not met. Thus, this review mirrors the lack of quantity of the included trials. The results of ongoing and future well designed, large randomized clinical trials are needed.

## Conclusions

Compared with DP-DES, O-SES showed a significantly reduced risk of MI and a trend toward reduction in ST.

## Appendix 1

**Table 3:** Search strategy on PubMed

#1	"Percutaneous Coronary Intervention"[Mesh]
#2	"Coronary Disease"[Mesh]
#3	"PCI"
#4	"CAD"
#5	(#1) OR (#2) OR (#3) OR (#4)
#6	"biodegradable"[tiab]
#7	"degradable"[tiab]
#8	"bioabsorbable"[tiab]
#9	"absorbable"[tiab]
#10	"absorptive"[tiab]
#11	" orsiro"[tiab]
#12	"O-SES"[tiab]
#13	"dissolvable"[tiab]
#14	(#6) OR (#7) OR (#8) OR (#9) OR (#10) OR (#11) OR (#12) OR (#13)
#15	"Polymers"[Mesh] OR "Polymer"[tiab] OR "coating"[tiab]
#16	(#14) AND (#15)
#17	"BioMatrix" OR "NOBORI" OR "Axxess" OR "Supralimus" OR "Infinnium" OR "BioMime" OR "Orsiro" OR "DESyne" OR "SYNERGY" OR "MiStent" OR "Excel" OR "Firehawk" OR "NOYA" OR "Inspiron" OR "Tivoli" OR "BuMA" OR "Svelte" OR "Custom" OR "NEVO" OR "Elixir" OR "JACTAX" OR "CORACTO"
#18	(#16)) OR (#17)
#19	"randomized controlled trial"[pt] OR "controlled clinical trial"[pt] OR "randomized"[tiab] OR "randomly"[tiab] OR "trial"[tiab] OR " clinical trials as topic"[sh]
#20	# (5) AND # (18) AND # (19)

## Appendix 2

**Table 4:** Characteristics of patients in eligible studies

	Age	Male sex (%)	Hypertension (%)	Diabetes mellitus (%)	Smoker (%)	Previous MI (%)	ACS (%)	Stable angina (%)
BIO-RESORT	64 ± 11	72	46	18	30	19	70	30
BIOFLOW II	63 ± 10	77	78	28	27	27	NR	NR
BIOFLOW V	65 ± 10	74	80	35	23	27	51	48
BIOSCIENCE	66 ± 12	77	68	23	29	20	53	31
ORIENT	65 ± 11	72	65	26	27	NR	45	55
PRISON IV	63 ± 10	78	56	20	33	30	17	70

## Appendix 3

**Table 5:** Primary and second outcomes of the Included Trials

	Primary outcome	Second outcomes
BIO-RESORT	target vessel failure at 12 months	target lesion failure, death, myocardial infarction, coronary revascularization, major adverse cardiac events, patient-oriented composite endpoint, definite or probable stent thrombosis
BIOFLOW II	in-stent late lumen loss at 9 months	in-segment late lumen loss and in-stent and in-segment minimal luminal diameter, percent diameter stenosis, and binary restenosis. Cardiac death, procedure-related deaths, myocardial infarction, target-lesion revascularization.
BIOFLOW V	target lesion failure at 12 months	major adverse cardiac events (all-cause death, myocardial infarction or ischemia-driven target lesion revascularization), target vessel failure, the individual components of the composite endpoints at 30 days and 12 months, and definite or probable stent thrombosis according to academic research consortium (arc) criteria.
BIOSCIENCE	target-lesion failure at 12 months	all-cause death, cardiac death, myocardial infarction, target vessel mi, coronary revascularization, major adverse cardiac events, patient-oriented composite endpoint, stent thrombosis, target lesion revascularization, target vessel revascularization, repeat revascularization, target vessel failure, cerebrovascular event
ORIENT	in-stent late lumen loss at 9 months,	in-segment late lumen loss, percentage diameter stenosis, and binary restenosis at 9 months; all-cause death, cardiac death, myocardial infarction, repeat revascularization, ischemic stroke, hemorrhagic stroke, bleeding, stent thrombosis, target lesion failure, target vessel failure
PRISON IV	in-segment late lumen loss at 9 months	in-stent late lumen loss, in-stent and in-segment percentage of diameter stenosis, binary restenosis, and re-occlusions at 9 months; clinically indicated target lesion, revascularization or target vessel revascularization, myocardial infarction, death (cardiac and noncardiac), stent thrombosis, target vessel failure, and major adverse cardiac events.

## Abbreviations

BP: Biodegradable polymer
CI: Confidence intervals
DES: Drug-eluting stent
DP: Durable polymer
EES: Everolimus-eluting stents
MI: Myocardial infarction
O-SES: Orsiro biodegradable polymer sirolimus-eluting stent
RCTs: Randomized controlled trials
RR: Risk ratios
ST: Stent thrombosis
TLR: Target lesion revascularization
TVR: Target vessel revascularization
ZES: Zotarolimus-eluting stents

## References

[1] Byrne RA, Stone GW, Ormiston J, Kastrati A (2017). Coronary balloon angioplasty, stents, and scaffolds. Lancet.

[2] Joner M, Finn AV, Farb A, Mont EK, Kolodgie FD, Ladich E, Kutys R, Skorija K, Gold HK, Virmani R (2006). Pathology of drug-eluting stents in humans: delayed healing and late thrombotic risk. J Am Coll Cardiol.

[3] Finn AV, Nakazawa G, Joner M, Kolodgie FD, Mont EK, Gold HK, Virmani R (2007). Vascular responses to drug eluting stents: importance of delayed healing. Arterioscler Thromb Vasc Biol.

[4] Navarese EP, Tandjung K, Claessen B, Andreotti F, Kowalewski M, Kandzari DE, Kereiakes DJ, Waksman R, Mauri L, Meredith IT (2013). Safety and efficacy outcomes of first and second generation durable polymer drug eluting stents and biodegradable polymer biolimus eluting stents in clinical practice: comprehensive network meta-analysis. BMJ.

[5] Kang SH, Park KW, Kang DY, Lim WH, Park KT, Han JK, Kang HJ, Koo BK, Oh BH, Park YB (2014). Biodegradable-polymer drug-eluting stents vs. bare metal stents vs. durable-polymer drug-eluting stents: a systematic review and Bayesian approach network meta-analysis. Eur Heart J.

[6] Palmerini T, Benedetto U, Biondi-Zoccai G, Della Riva D, Bacchi-Reggiani L, Smits PC, Vlachojannis GJ, Jensen LO, Christiansen EH, Berencsi K (2015). Long-term safety of drug-eluting and bare-metal stents: evidence from a comprehensive network meta-analysis. J Am Coll Cardiol.

[7] Stefanini GG, Holmes DR (2013). Drug-eluting coronary-artery stents. N Engl J Med.

[8] El-Hayek G, Bangalore S, Casso Dominguez A, Devireddy C, Jaber W, Kumar G, Mavromatis K, Tamis-Holland J, Samady H (2017). Meta-analysis of randomized clinical trials comparing biodegradable polymer drug-eluting stent to second-generation durable polymer drug-eluting stents. JACC Cardiovasc Interv.

[9] Stefanini GG, Taniwaki M, Windecker S (2014). Coronary stents: novel developments. Heart.

[10] Kolandaivelu K, Swaminathan R, Gibson WJ, Kolachalama VB, Nguyen-Ehrenreich KL, Giddings VL, Coleman L, Wong GK, Edelman ER (2011). Stent thrombogenicity early in high-risk interventional settings is driven by stent design and deployment and protected by polymer-drug coatings. Circulation.

[11] Kastrati A, Mehilli J, Dirschinger J, Dotzer F, Schuhlen H, Neumann FJ, Fleckenstein M, Pfafferott C, Seyfarth M, Schomig A (2001). Intracoronary stenting and angiographic results: strut thickness effect on restenosis outcome (ISAR-STEREO) trial. Circulation.

[12] Windecker S, Stortecky S, Stefanini GG, da Costa BR, Rutjes AW, Di Nisio M, Silletta MG, Maione A, Alfonso F, Clemmensen PM (2014). Revascularisation versus medical treatment in patients with stable coronary artery disease: network meta-analysis. BMJ.

[13] Windecker S, Haude M, Neumann FJ, Stangl K, Witzenbichler B, Slagboom T, Sabate M, Goicolea J, Barragan P, Cook S (2015). Comparison of a novel biodegradable polymer sirolimus-eluting stent with a durable polymer everolimus-eluting stent: results of the randomized BIOFLOW-II trial. Circ Cardiovasc Interv.

[14] von Birgelen C, Kok MM, van der Heijden LC, Danse PW, Schotborgh CE, Scholte M, Gin R, Somi S, van Houwelingen KG, Stoel MG (2016). Very thin strut biodegradable polymer everolimus-eluting and sirolimus-eluting stents versus durable polymer zotarolimus-eluting stents in allcomers with coronary artery disease (BIO-RESORT): a three-arm, randomised, non-inferiority trial. Lancet.

[15] Zbinden R, Piccolo R, Heg D, Roffi M, Kurz DJ, Muller O, Vuilliomenet A, Cook S, Weilenmann D, Kaiser C (2016). Ultrathin strut biodegradable polymer Sirolimus-eluting stent versus durable-polymer Everolimus-eluting stent for percutaneous coronary revascularization: 2-year results of the BIOSCIENCE trial. J Am Heart Assoc.

[16] Kandzari DE, Mauri L, Koolen JJ, Massaro JM, Doros G, Garcia-Garcia HM, Bennett J, Roguin A, Gharib EG, Cutlip DE (2017). Ultrathin, bioresorbable polymer sirolimus-eluting stents versus thin, durable polymer everolimus-eluting stents in patients undergoing coronary revascularisation (BIOFLOW V): a randomised trial. Lancet.

[17] Kang SH, Chung WY, Lee JM, Park JJ, Yoon CH, Suh JW, Cho YS, Doh JH, Cho JM, Bae JW (2017). Angiographic outcomes of Orsiro biodegradable polymer sirolimus-eluting stents and resolute integrity durable polymer zotarolimus-eluting stents: results of the ORIENT trial. EuroIntervention.

[18] Teeuwen K, van der Schaaf RJ, Adriaenssens T, Koolen JJ, Smits PC, Henriques JP, Vermeersch PH, Joe T, Gin RM, Scholzel BE, Kelder JC (2017). Randomized multicenter trial investigating angiographic outcomes of hybrid Sirolimus-eluting stents with biodegradable polymer compared with Everolimus-eluting stents with durable polymer in chronic Total occlusions: the PRISON IV trial. JACC Cardiovasc Interv.

[19] Liberati A, Altman DG, Tetzlaff J, Mulrow C, Gotzsche PC, Ioannidis JP, Clarke M, Devereaux PJ, Kleijnen J, Moher D (2009). The PRISMA statement for reporting systematic reviews and meta-analyses of studies that evaluate healthcare interventions: explanation and elaboration. BMJ.

[20] Shinichi A (2014). Cochrane handbook for systematic reviews of interventions. Online Kensaku.

[21] Guyatt GH, Oxman AD, Vist GE, Kunz R, Falck-Ytter Y, Alonso-Coello P, Schunemann HJ (2008). GRADE: an emerging consensus on rating quality of evidence and strength of recommendations. BMJ.

[22] Higgins JP, Thompson SG (2002). Quantifying heterogeneity in a meta-analysis. Stat Med.

[23] Brok J, Thorlund K, Gluud C, Wetterslev J (2008). Trial sequential analysis reveals insufficient information size and potentially false positive results in many meta-analyses. J Clin Epidemiol.

[24] Pandya B, Gaddam S, Raza M, Asti D, Nalluri N, Vazzana T, Kandov R, Lafferty J (2016). Biodegradable polymer stents vs second generation drug eluting stents: a meta-analysis and systematic review of randomized controlled trials. World J Cardiol.

[25] Yang Y, Lei J, Huang W, Lei H (2016). Efficacy and safety of biodegradable polymer sirolimus-eluting stents versus durable polymer drug-eluting stents: a meta-analysis of randomized trials. Int J Cardiol.

[26] Soucy NV, Feygin JM, Tunstall R, Casey MA, Pennington DE, Huibregtse BA, Barry JJ (2010). Strut tissue coverage and endothelial cell coverage: a comparison between bare metal stent platforms and platinum chromium stents with and without everolimus-eluting coating. EuroIntervention.

[27] Pache J, Kastrati A, Mehilli J, Schuhlen H, Dotzer F, Hausleiter J, Fleckenstein M, Neumann FJ, Sattelberger U, Schmitt C (2003). Intracoronary stenting and angiographic results: strut thickness effect on restenosis outcome (ISAR-STEREO-2) trial. J Am Coll Cardiol.

[28] Kandzari DE, Leon MB, Popma JJ, Fitzgerald PJ, O’Shaughnessy C, Ball MW, Turco M, Applegate RJ, Gurbel PA, Midei MG (2006). Comparison of zotarolimus-eluting and sirolimus-eluting stents in patients with native coronary artery disease: a randomized controlled trial. J Am Coll Cardiol.

[29] Stone GW, Midei M, Newman W, Sanz M, Hermiller JB, Williams J, Farhat N, Mahaffey KW, Cutlip DE, Fitzgerald PJ (2008). Comparison of an everolimus-eluting stent and a paclitaxel-eluting stent in patients with coronary artery disease: a randomized trial. Jama.

[30] Leon MB, Mauri L, Popma JJ, Cutlip DE, Nikolsky E, O’Shaughnessy C, Overlie PA, McLaurin BT, Solomon SL, Douglas JS (2010). A randomized comparison of the Endeavor zotarolimus-eluting stent versus the TAXUS paclitaxel-eluting stent in de novo native coronary lesions 12-month outcomes from the ENDEAVOR IV trial. J Am Coll Cardiol.

[31] Stone GW, Rizvi A, Newman W, Mastali K, Wang JC, Caputo R, Doostzadeh J, Cao S, Simonton CA, Sudhir K (2010). Everolimus-eluting versus paclitaxel-eluting stents in coronary artery disease. N Engl J Med.

[32] Bangalore S, Toklu B, Amoroso N, Fusaro M, Kumar S, Hannan EL, Faxon DP, Feit F (2013). Bare metal stents, durable polymer drug eluting stents, and biodegradable polymer drug eluting stents for coronary artery disease: mixed treatment comparison meta-analysis. BMJ.

[33] Cassese S, Byrne RA, Ndrepepa G, Kufner S, Wiebe J, Repp J, Schunkert H, Fusaro M, Kimura T, Kastrati A (2016). Everolimus-eluting bioresorbable vascular scaffolds versus everolimus-eluting metallic stents: a meta-analysis of randomised controlled trials. Lancet.

